# Human thymic putative CD8αα precursors exhibit a biased TCR repertoire in single cell AIRR-seq

**DOI:** 10.1038/s41598-023-44693-4

**Published:** 2023-10-18

**Authors:** Marte Heimli, Siri Tennebø Flåm, Hanne Sagsveen Hjorthaug, Pål Marius Bjørnstad, Maria Chernigovskaya, Quy Khang Le, Xavier Tekpli, Victor Greiff, Benedicte Alexandra Lie

**Affiliations:** 1 Department of Medical Genetics, University of Oslo and Oslo University Hospital, 0424 Oslo, Norway; 2 Department of Immunology, University of Oslo and Oslo University Hospital, 0372 Oslo, Norway

**Keywords:** VDJ recombination, Immune tolerance

## Abstract

Thymic T cell development comprises T cell receptor (TCR) recombination and assessment of TCR avidity towards self-peptide-MHC complexes presented by antigen-presenting cells. Self-reactivity may lead to negative selection, or to agonist selection and differentiation into unconventional lineages such as regulatory T cells and CD8$\mathrm{\alpha \alpha }$ T cells. To explore the effect of the adaptive immune receptor repertoire on thymocyte developmental decisions, we performed single cell adaptive immune receptor repertoire sequencing (scAIRR-seq) of thymocytes from human young paediatric thymi and blood. Thymic *PDCD1*^+^ cells, a putative CD8$\mathrm{\alpha \alpha }$ T cell precursor population, exhibited several TCR features previously associated with thymic and peripheral *ZNF683*^+^ CD8$\mathrm{\alpha \alpha }$ T cells, including enrichment of large and positively charged complementarity-determining region 3 (CDR3) amino acids. Thus, the TCR repertoire may partially explain the decision between conventional vs. agonist selected thymocyte differentiation, an aspect of importance for the development of therapies for patients with immune-mediated diseases.

## Introduction

Diversity of the adaptive immune cell receptor repertoire (AIRR) is ensured by genetic recombination of Variable (V), Joining (J), and Diversity (D) gene segments^1,2^. During thymic T cell development, V(D)J recombination of $\upbeta ,\upgamma , \mathrm{and}\,\updelta$ T cell receptor (TCR) chains occurs at the CD4^−^CD8^−^ double-negative (DN) stage, while TCR$\mathrm{\alpha }$ chain recombination occurs later at the CD4^+^CD8^+^ double-positive (DP) stage^1,3^. TCR recombination is coupled to selection checkpoints in order to restrict maturation to thymocytes bearing in-frame, functionally recombined, TCRs without self-reactive capabilities^4^. Despite the selection processes, a fraction of self-reactive thymocytes escape to the periphery, conferring risk of autoimmune responses^5,6^.

Escaped self-reactive T cells may be held in check by unconventional, agonist selected T cell populations such as regulatory T cells (T_regs_)^7^. Agonist selected populations are of high interest due to their immune-modulating functions and therapeutic potential^8–11^, however, knowledge regarding thymic heterogeneity and development of agonist selected T cells remains incomplete^12–14^. In similarity to developing T_regs_, thymic precursors of CD8$\mathrm{\alpha \alpha }$^+^ intraepithelial lymphocytes (IELs) have been suggested to undergo agonist selection^15,16^.

Previously, we have studied gene expression (GEX) in cell populations in young paediatric thymi, in order to explore the influence of the local cellular milieu for thymic agonist selection^17^. Since the TCR could also play a role in determining divergence towards an agonist-selected T cell fate, we here performed single cell AIRR sequencing (scAIRR-seq) to assess the TCR repertories of the same cells. The current results indicated changes in the TCR repertoire during developmental progression of pre-selection DP thymocytes. In addition, we found biases in the TCR repertoire for a thymic *PDCD1*^+^ putative CD8$\mathrm{\alpha \alpha }$ T cell precursor population that have previously been described for *ZNF683*^+^ CD8$\mathrm{\alpha \alpha }$ T cells. Our results add insights regarding how the TCR influence decision checkpoints during thymocyte development, in particular the decision between agonist versus conventional selection.

## Methods

### Study population

Thymic tissues and EDTA blood samples were collected from young paediatric donors (3 male, 2 female, age span 7 days–13.5 months) undergoing corrective heart surgery at the Department of Cardiothoracic surgery, Oslo University Hospital (Supplementary table 1). The project was approved by the Regional Ethics committee of South East Norway (REC 31516) and conducted in compliance with the Declaration of Helsinki. Written informed consent was obtained from donor parents.

### Sample processing

Sample collection and processing has been described previously^17^. Briefly, thymic tissue was dissociated by mechanical and enzymatic (Liberase TM) treatment. For each donor (N = 5), four samples were profiled: (1) Peripheral blood mononuclear cells (PBMC), (2) thymic cells without enrichment, (3) thymic cells enriched for APCs by density gradient centrifugation, and (4) CD45-depleted thymic cells.

### Single cell AIRR sequencing

Single cell sequencing was performed according to the 10× Genomics Chromium Single Cell 5′ V(D)J Reagent Kit User guide with Feature Barcode Technology for Cell Surface Protein, v1 Chemistry (10× Genomics protocol CG000186, RevD). Amplification of full length cDNA prepared from mRNA was run for 13 cycles for PBMC and unenriched samples, and 15 cycles for APC-enriched and CD45-depleted samples, before single cell TCR V(D)J enriched libraries were constructed. In brief, 2 $\upmu$l cDNA was used to enrich full-length V(D)J segments via PCR amplification with primers specific to the $\mathrm{\alpha \beta }$TCR, before enzymatic fragmentation and size selection by beads. Indexing PCR was run for 9 cycles for all samples as per manufacturer’s recommendation, and sequencing was performed on an Illumina NovaSeq S2 flow cell (read length 27 for R1, 92 for R2).

### Pre-processing and filtering of single cell AIRR data

Single cell AIRR sequencing data was pre-processed using CellRanger v.3.1.0 with alignment to the refdata-cellranger-vdj-GRCh38-alts-emsebl-5.0.0 reference^18,19^. Most libraries reached a sequencing depth of > 5000 read pairs/cell, with two exceptions for the CD45-depleted TCR library for donor 3 (3300 read pairs/cell) and the APC-enriched TCR library for donor 2 (4353 read pairs/cell). Clonotypes were defined as by CellRanger v.3.1.0, according to exact match of the complementarity-determining region 3 (CDR3) nucleotide sequence. Alternatively, for analyses focusing on the CDR3 amino acid sequence, we defined clonotypes by exact match in CDR3 amino acid (CDR3aa) sequence.

Further analysis (except CoNGA) was performed in R v.4.1.3^20^ using Immunarch v.0.8.0^21^. The filtered_contig_annotation.csv files from CellRanger were loaded as paired chain data by use of repLoad() with .mode = “paired”, and barcodes annotated as thymocytes/T cells in the GEX dataset were retained. Next, CellRanger-derived clonotypes were filtered by chain combinations, permitting a) a single *TRB* chain or a pair of one *TRA* and one *TRB* for thymocytes, and b) a pair of one *TRA* and one *TRB* for peripheral blood T cells.

Downstream analyses in Immunarch were performed using single receptor chains, loaded by repLoad() with .mode = “single”. The resulting dataset was filtered by keeping barcodes that existed in filtered, paired chain data, and grouped by GEX data cell type annotations.

For calculation of numbers and abundances of unique CDR3aa sequences in the filtered single chain data, identical CDR3aa sequences were combined and their counts summed up. Unique CDR3aa numbers were calculated using repExplore() with .method = “volume” and .col = “aa”. The abundance of unique CDR3aa sequences was calculated using repExplore() with .method = “count” and .col = “aa”.

### CDR3 lengths, clonal overlap, and gene usage in Immunarch

CDR3aa lengths were calculated for thymic TCRs by use of single chain data, after combining data from all samples for each cell type. For thymocytes, cell types were further grouped as early stage (DN(P), DN(Q), DP(P), DP(Q)), late stage ($\alpha \beta$T(entry), CD4^+^ SP, CD8^+^ SP) or agonist (CD8$\mathrm{\alpha \alpha }$(I), CD8$\mathrm{\alpha \alpha }$(II), T_(agonist)_, T_reg_(diff), T_reg_) thymocytes. Identical CDR3aa sequences were combined and summed up before running repExplore() with .method = “len” and .col = “aa”. A two-tailed Welsh t-test was performed between early stage thymocytes and either late stage or agonist thymocytes, with a null hypothesis of no difference in group CDR3aa length means (H_0_: ${\mu }_{1}= {\mu }_{2})$, an alternative hypothesis of a difference in group CDR3aa length means (H_a_: ${\mu }_{1}\ne {\mu }_{2})$, and an alpha level of 0.05, followed by Bonferroni correction for two tests.

Clonal overlap was evaluated for singe chain data, after combining data from all samples by either cell type or donor, and grouping and summing up identical CDR3aa sequences. Overlap was determined by the Morisita-Horn overlap index, which accounts for both numbers and abundances of overlapping clones^22,23^. For this, repOverlap() was used with .method = “morisita” and .col = “aa”.

Gene usage was determined based on single chain data combined by cell type, according to CellRanger-derived clonotype definitions. One clonotype lacking *TRAJ* gene information and one clonotype lacking *TRBJ* gene information were excluded. Weighted gene usage was calculated as proportions weighted by the abundance of each clonotype, using the Immunarch geneUsage() function with .quant = “count”, .norm = T, and .ambig = “maj”.

### Overlap of the TCR dataset to McPAS-TCR

For identification of pathology-specific TCR sequences, we assessed our data for overlap to the McPAS-TCR database (accessed 02.02.2023), which contains pathology-associated *TRA* and *TRB* CDR3aa sequences with known antigen specificities^24^. Human sequences under the “Autoimmune” and “Pathogens” categories of McPAS-TCR were extracted, encompassing 26258 *TRB* sequences and 6999 *TRA* sequences. We then tested for overlap to the database among unique, single chain CDR3aa *TRA* or *TRB* sequences for early stage thymocytes, late stage thymocytes, agonist thymocytes, peripheral CD4 T cells (CD4 cytotoxic T cell (CTL), CD4 naive, CD4 Proliferating, CD4 T central memory (TCM), CD4 T effector memory (TEM)), and peripheral CD8 T cells (CD8 naive, CD8 TCM, CD8 TEM). Enrichment of pathology-specific sequences among cell groups relative to the database was assessed by a one-tailed Fisher’s exact t-test (H_0_: true odds ratio = 1, H_A_: true odds ratio > 1) with Bonferroni correction for the number of tested diseases and the number of tested cell type groups, based on the approach by Amoriello et al.^25^.

### Joint TCR and transcriptomic analysis using CoNGA

We performed clonotype neighbour graph analysis (CoNGA)^26^, a joint analysis pipeline for TCR and GEX data in order to identify correlations between the data types. The CoNGA pipeline includes creation of separate neighbourhood graphs for clonotypes in the TCR and GEX datasets, followed by identification of clonotypes that reside within overlapping neighbourhoods in both datasets. For CoNGA analysis (commit ID b572f73e5c90aaae59e11f187553a50324f26f02) of either thymocytes or peripheral T cells, filtered, paired chain TCR data was used, and analysed together with previously reported GEX data for either thymocytes or T cells.

After log normalization and then regression in order to account for the effect of number of counts and percentage of mitochondrial genes in the GEX data, data was reduced to one representative cell per CellRanger-derived clonotype. Neighbourhood graphs were created for the GEX and TCR datasets by use of BBKNN v. 1.5.1^27^, before UMAP dimensionality reduction^28^ and clustering by the Leiden algorithm^29^ at resolution 1.0. Differential gene expression analysis was performed by the Wilcoxon Rank Sum test by the rank_genes_group() function from Scanpy v. 1.9.1, as implemented by the CoNGA find_gex_cluster_degs() function.

Next, the CoNGA graph-versus-graph analysis was used, which calculates a “CoNGA score” for identification of clonotypes residing within overlapping neighbourhoods in GEX and TCR datasets, and combines overlapping clonotypes into “CoNGA clusters”. This was done by the CoNGA run_graph_vs_graph() function, with a minimum size for identified “CoNGA clusters” of 22 clonotypes for the thymocyte dataset, and 8 for the PBMC-derived T cell dataset, constituting at least 0.1% of clonotypes in the dataset.

Graph-versus-feature analysis was used for identification of TCR features exhibiting variation across the GEX dataset, by running the CoNGA run_graph_vs_feature() function. Reported adjusted P-values are from assessment of variation in TCR features across a GEX cluster graph, where clonotypes residing in the same GEX cluster are connected. As implemented in CoNGA in order to increase computational speed, features were first subjected to a preliminary t-test, and p-values were adjusted by multiplication for the number of performed tests. Features with multiplied p-values $\le$ 10 were then subjected to a two-tailed Mann–Whitney U test with Bonferroni correction, at an alpha level of 0.05.

### Visualisations

Visualisations were prepared as implemented in Immunarch or CoNGA, in addition to ggplot2 v.3.3.5^30^.

## Results

### Sample preparation and data filtering

**Figure 1:**
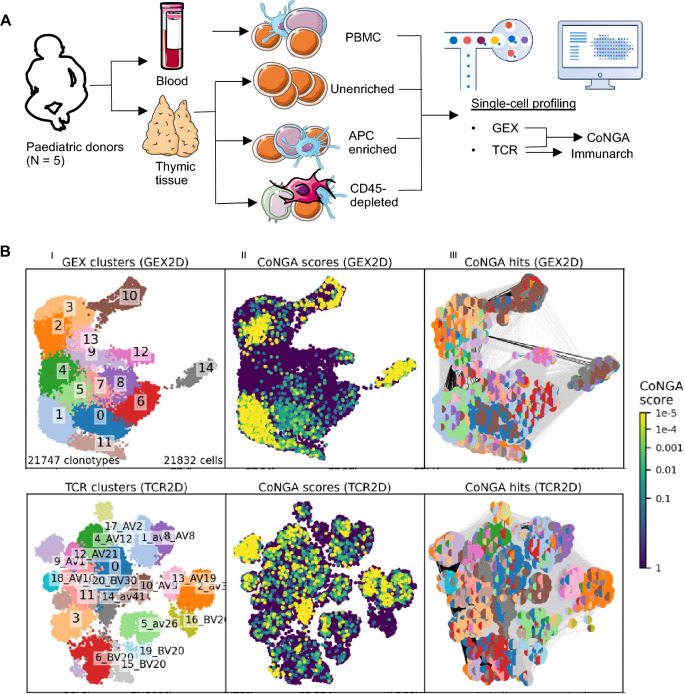
Single cell gene expression (GEX) and adaptive immune receptor repertoire (AIRR) sequencing of young paediatric (N = 5) thymi and blood. (**A**) Experimental set-up. For each donor, single cell AIRR sequencing was performed for one PBMC sample and three thymic samples. Created using content from Servier Medical Art, provided by Servier, licensed under a Creative Commons Attribution 3.0 unported license, and graphics adapted from 10 × Genomics. (**B**) Clonotype Neighbour Graph Analysis (CoNGA) for identification of correlations between thymocyte GEX and T cell receptor (TCR) datasets. (I) Clonotypes that are highly similar in either the GEX (top row) or TCR (bottom row) datasets, based on neighbourhood graphs, are clustered together. (II) Groups of clonotypes with similar GEX and TCR profiles are identified as having low “CoNGA scores”. The “CoNGA scores” reflect the likelihood of seeing equal or greater overlap between the GEX and TCR dataset by chance, when overlap is assessed as shared neighbours between the two datasets for each clonotype. (III) Clonotypes with low “CoNGA scores” (“CoNGA hits”) are grouped into “CoNGA clusters”, bi-coloured discs indicate their GEX (left half) and TCR (right half) cluster assignment.

To study TCR repertoires of human thymus and blood, scAIRR-seq was performed. Briefly, one PBMC sample and three thymic samples were prepared for each young paediatric donor (N = 5), with thymic samples consisting of one unenriched sample, one sample enriched for APCs, and one CD45-depleted sample (Fig. 1A). Single cell TCR sequencing was performed for all 20 samples.

AIRR data was filtered to retain immune receptor chains from thymocytes/T cells based on previous annotations^17^, and presenting in expected chain combinations (*TRB* or *TRA* + *TRB* for thymocytes, *TRA* + *TRB* for peripheral T cells). This resulted in 46086 unique cell barcodes in the TCR data. The number of unique CDR3aa sequences for each chain was proportional to cell numbers and cell composition of samples, with most abundant cell types having higher numbers of unique CDR3aa sequences (Supplementary Fig. 1A). For each chain, the clonal abundance was low, with most unique CDR3aa sequences being associated with one single cell per sample (Supplementary Fig. 1B).

### Joint TCR and transcriptomic analysis confirms previously identified thymocyte populations

In order to determine whether changes in TCR repertoires during the course of thymocyte developmental progression could be observed, we used two approaches for data analysis: One based on Immunarch using our previously reported annotations, and one based on the CoNGA pipeline^26^. The CoNGA pipeline performs a joint analysis of both GEX and AIRR data for identification of correlations between both data types to enable new discoveries not revealed by the GEX data alone.

**Figure 2:**
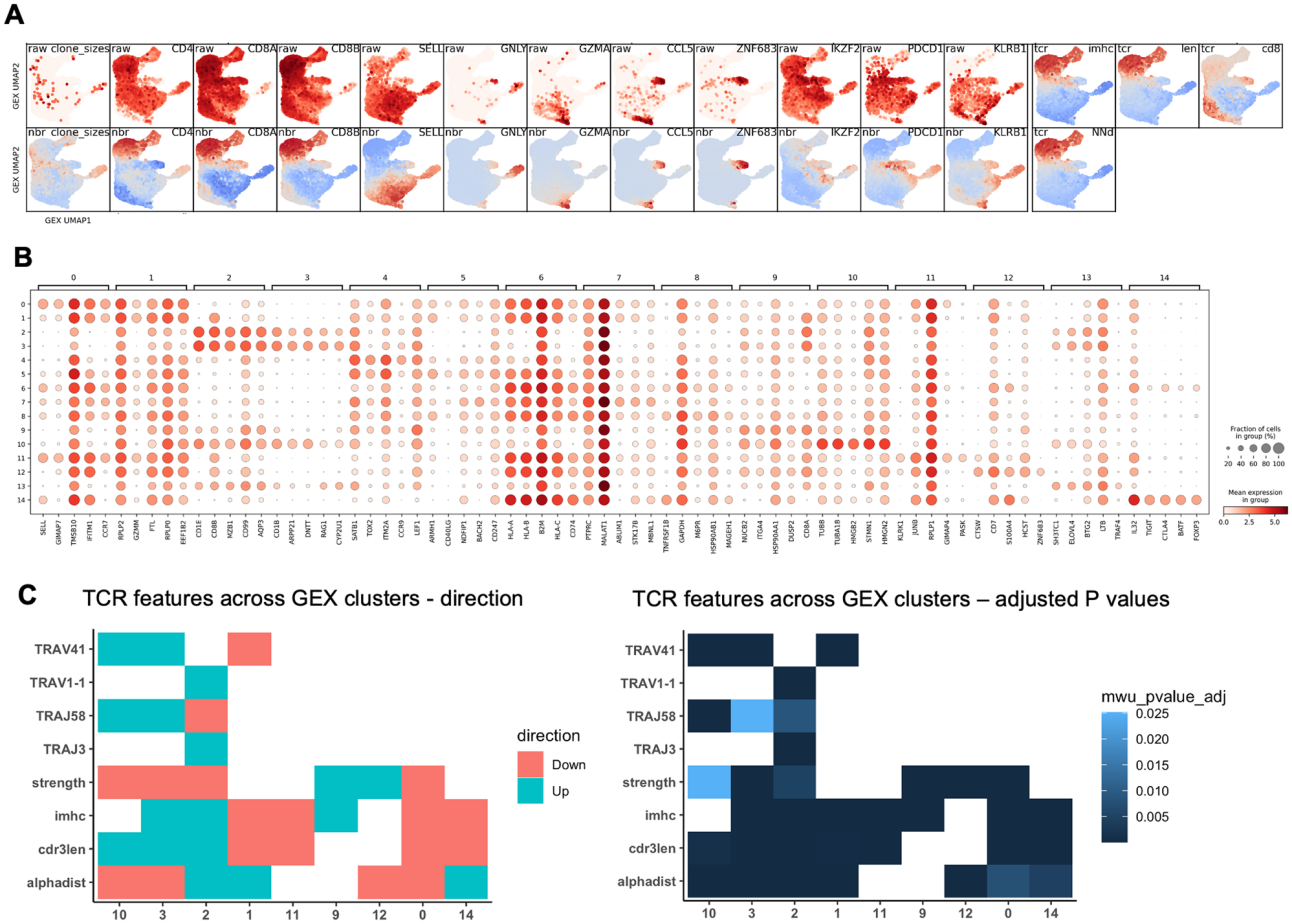
Gene expression and T cell receptor (TCR) features across single cell gene expression (GEX) defined clusters. (**A**) Selected genes and TCR features across the thymic GEX UMAP. Colour indicates expression of selected genes (top row) and Z-score normalised and GEX-neighbourhood averaged expression of the same genes (bottom row). Blue colour indicates negative values, red colour indicates positive values. (**B**) Differentially expressed genes across thymic GEX clusters. Dot colour indicates expression level, and dot size indicates the fraction of clonotypes expressing the gene. (**C**) Selected TCR features exhibiting variation across GEX clusters in a graph-versus-feature analysis. Numerical features indicating properties of the TCR repertoire are mapped onto a GEX cluster graph where clonotypes residing in the same GEX cluster are connected. Variation in the TCR features across the GEX cluster graph is assessed first by an initial t-test, then by a one-sided Mann–Whitney U test for the features with low t-test P values. Colours indicate direction of change (left) or adjusted Mann–Whitney U test P values (right).

In the CoNGA-generated GEX UMAP (Figs. 1B, 2A, B Supplementary Fig. 2A), populations previously reported by us and others were identified^14^, including proliferating (P) DP thymocytes (GEX cluster 10), quiescent (Q) DP thymocytes (GEX clusters 3 and 2), CD8^+^ SP thymocytes (GEX clusters 1 and 11), and CD4^+^ SP thymocytes (GEX cluster 0 among others). GEX cluster 11 contained two distinguishable subsets, where one expressed genes associated with an innate-like phenotype, in resemblance to the population termed NKT cells in our previous report. We also observed clusters resembling *ZNF683*^+^ CD8$\mathrm{\alpha \alpha }$ cells (GEX cluster 12) and *FOXP3*^+^ T_regs_ (GEX cluster 14), and a cluster (GEX cluster 9) expressing *PDCD1* in similarity to the thymic Type A CD8$\alpha \alpha$ precursor^15,31^.

### A shift from proximal to distal TRA gene usage occurs at the DP(Q) thymocyte stage

We next investigated potential differences in TCR gene usage between thymocyte populations. In the CoNGA pipeline, clonotypes that reside within overlapping neighbourhoods in the GEX and TCR datasets are combined into “CoNGA clusters” (GEX:TCR) (Fig. 1B, Supplementary Fig. 3). One “CoNGA cluster” (10:14) contained clonotypes from the DP(P) GEX cluster 10 and exhibited high usage of the proximal *TRAV41* gene. DP(Q) thymocytes in GEX clusters 2 and 3 participated in several distinct “CoNGA clusters”, including one (3:14) biased towards *TRAV41*, and one (2:9) biased towards *TRAV1-2* and *TRAV1-1*.

To further identify variation in TCR features across the thymocyte GEX dataset, we used the CoNGA graph-versus-feature analysis (Fig. 2C, Supplementary Fig. 4, Supplementary table 5). Among the assessed TCR features, CoNGA implements numerical scores representing properties of the TCR, including scores previously developed by others^32,33^, and novel scores by the CoNGA developers^26^. For instance, the “alphadist” score represents the ordinal distance between the *TRAV* and *TRAJ* gene segments when the *TCRA* locus is ordered by genomic position^26^. The “alphadist” score was significantly decreased for GEX clusters 10 (DP(P) thymocytes, P_adj_ = 4.3 × 10^–96^) and 3 (DP(Q) thymocytes, P_adj_ = 7.0 × 10^–80^), and significantly increased for GEX cluster 2 (DP(Q) thymocytes, P_adj_ = 9.0 × 10^–97^), fitting with the gene usage patterns noted above.

**Figure 3:**
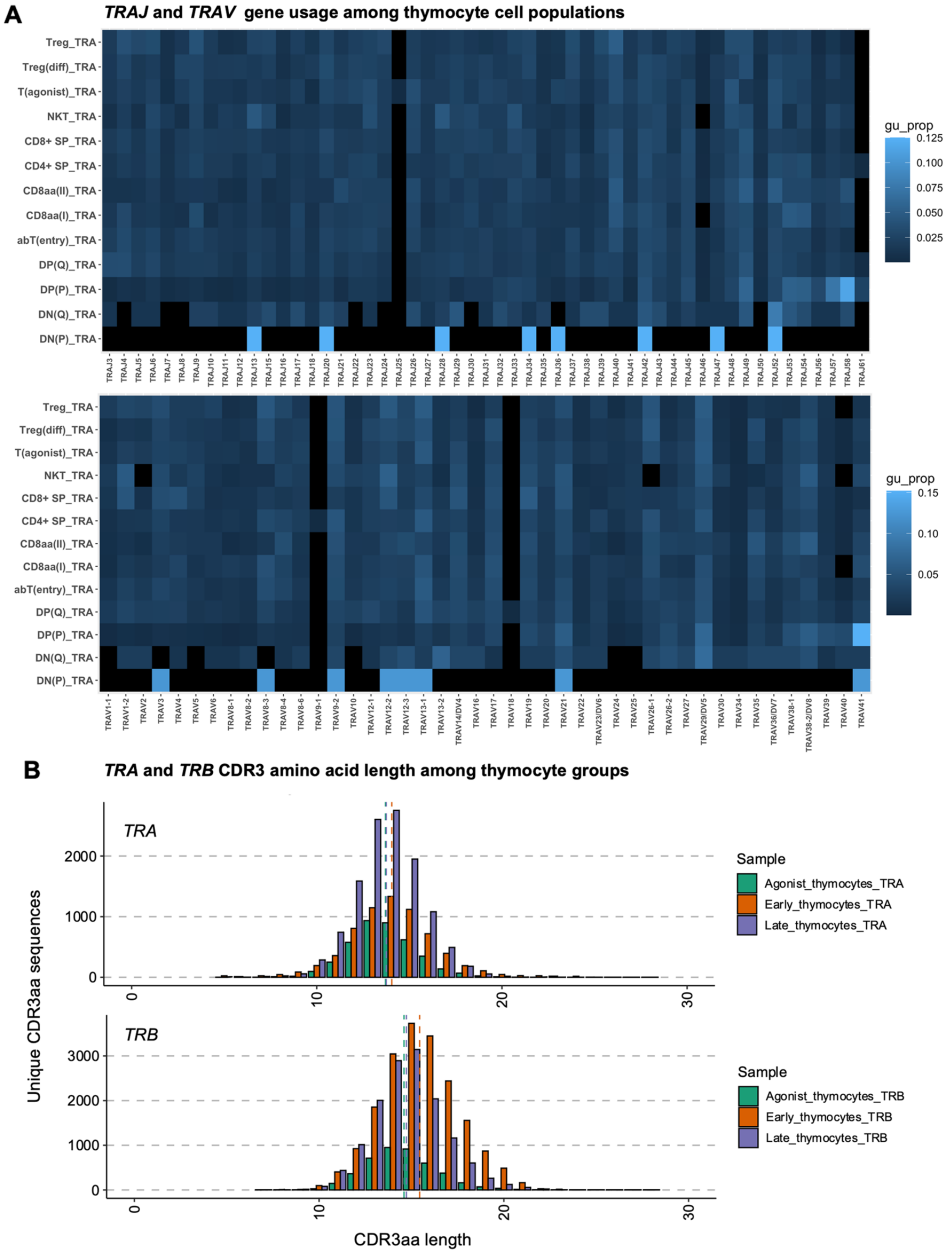
Gene usage proportions and distribution of CDR3aa lengths among thymocytes. (**A**) *TRAJ* and *TRAV* gene usage across thymocytes. Colour indicates proportion among CellRanger-derived clonotypes weighted by clonal counts. (**B**) Length of unique *TRA* and *TRB* CDR3aa sequences among agonist (CD8 $\mathrm{\alpha \alpha }$(I), CD8$\mathrm{\alpha \alpha }$(II), T(agonist), T_reg_(diff), T_reg_), early (DN(P), DN(Q), DP(P), DP(Q)), and late ($\mathrm{\alpha \beta }$T(entry), CD4^+^ SP, CD8^+^ SP) thymocytes, dotted vertical lines indicate group means.

In agreement, analysis by Immunarch (Fig. 3A, Supplementary Fig. 5) revealed a high frequency of *TRAV41* for DP(P), in addition to a high frequency of *TRAJ57* and *TRAJ58*. We finally noted diversity in gene usage among the innate-like population labelled as Natural Killer T (NKT) cells, in agreement with reports of a CD8^+^ innate-like polyclonal thymic cell population from mice^34^, and NK-like CD8^+^ T cells in humans^35^. In sum, DP(P) thymocytes exhibited a bias toward proximal *TRA* genes, while shift from proximal to distal *TRA* gene usage occurred at the DP(Q) thymocyte stage.

### Early thymocytes have longer CDR3aa sequences compared to late or agonist thymocytes

We next investigated CDR3aa length across thymocytes. In the CoNGA graph-versus-feature analysis (Fig. 2C, Supplementary Fig. 4, Supplementary table 5), DP thymocytes (GEX clusters 10, 2, and 3) exhibited significantly increased CDR3aa lengths (P_adj_ = 0.0011 for GEX cluster 10, 7.7 × 10^–61^ for GEX cluster 2, 2.2 × 10^–48^ for GEX cluster 3) compared to remaining clusters. By contrast, CDR3aa length was significantly decreased for the more mature CD8^+^ (GEX cluster 1, P_adj_ = 3.0 × 10^–4^ and GEX cluster 11, P_adj_ = 8.1 × 10^–6^) and CD4^+^ SP (GEX cluster 0, P_adj_ = 3.7 × 10^–7^) thymocyte stages.

To determine whether the same conclusion was supported by Immunarch, we grouped unique *TRA* or *TRB* CDR3aa sequences of early stage, late stage, and agonist selected thymocytes (Supplementary table 2). We tested for differences in CDR3aa length between early stage thymocytes and either late stage or agonist thymocytes by Welsh’s t-test, under an assumption of a normal distribution (Fig. 3B, Supplementary table 3). Early stage thymocytes exhibited significantly longer CDR3aa lengths compared to late stage thymocytes (P_adj_ = 1.7 × 10^–17^ for *TRA*, 7.1 × 10^–220^ for *TRB*) or agonist selected thymocytes (P_adj_ = 5.2 × 10^–16^ for *TRA*, 2.9 × 10^–138^ for *TRB*). In sum, both pipelines highlight a decrease in CDR3 length after the DP(Q) stage.

### *PDCD1*^+^ thymocytes exhibit previously reported *ZNF683*^+^-associated TCR biases

We further examined the potential biases in the TCR repertoire among thymocytes developing towards CD8$\mathrm{\alpha \alpha }$ T cells, as CoNGA identified specific biases for both thymic and PBMC *ZNF683*^**+**^ CD8$\mathrm{\alpha \alpha }$ T cells in a previous report^26^. While a “CoNGA cluster” (12:2) was formed by clonotypes from the *ZNF683*^+^ GEX cluster 12 and TCR cluster 2, the presence of additional 1:2 and 11:2 “CoNGA clusters” could indicate similar TCR features among other CD8^+^ T cells (Supplementary Fig. 3). However, the graph-versus-feature analysis indicated a significantly increased score for “strength” in GEX cluster 12 (P_adj_ = 2.5 × 10^–5^), a measure reflecting a CDR3aa composition mediating strong TCR interactions based on an estimated interaction potential^26,32,33,36^ (Fig. 2C, Supplementary Fig. 4, Supplementary table 5).

GEX cluster 12 (*ZNF683*^**+**^ CD8$\mathrm{\alpha \alpha }$) also exibited several features previously observed among thymic and peripheral *ZNF683*^+^ T cells^26^, including enrichement of large and positively charged amino acid residues (“volume” score, reflecting amino acid size, and “charge” score, reflecting amino acid charge^26,32,33^, Supplementary Fig. 4, Supplementary table 5). Still, no increase was observed in the score termed “independent of peptide:MHC” (“imhc”), which summarises the reported *ZNF683*^+^ T cell-associated TCR features as a weighted linear combination of several TCR sequence features, including “volume” and “charge” scores among others^26^.

By contrast, “imhc” was significantly upregulated for the *PDCD1-*expressing GEX cluster 9 (CD8$\mathrm{\alpha \alpha }$ precursor, P_adj_ = 3.3 × 10^–15^), and for the DP(Q) thymocytes in GEX clusters 3 (P_adj_ = 3.2 × 10^–35^) and 2 (P_adj_ = 1.18 × 10^–51^). Like GEX cluster 12, GEX cluster 9 was also significantly enriched for CDR3aa sequences mediating high affinity TCR interactions (”strength” score, P_adj_ = 1.3 × 10^–19^) (Fig. 2C, Supplementary Fig. 4, Supplementary table 5).

While T_reg_ progenitors mirroring the previously reported T_(agonist)_ and T_reg_(diff) populations were not identified in the CoNGA analysis, potentially as an effect of the employed cluster resolution, mature T_regs_ could be observed in GEX cluster 14 (Figs. 1B, 2B). This cluster exhibited reduced “imhc” and “charge” scores (Fig. 2C, Supplmentary table 5), thus constrasting with the observations from the CD8$\mathrm{\alpha \alpha }$ T cell lineage.

Overall, thymic *PDCD1*^+^ and *ZNF683*^+^ cells exhibited TCR features reported to be associated with *ZNF683*^+^ T cells. For the *PDCD1*^+^ cluster, this resulted in a significant upregulation of the summarising “imhc” score, suggesting a TCR repertoire bearing similarities to previously characterized *ZNF683*^+^ T cells.

### Peripheral *ZNF683*^+^ cells resemble their thymic counterparts with respect to TCR biases

**Figure 4:**
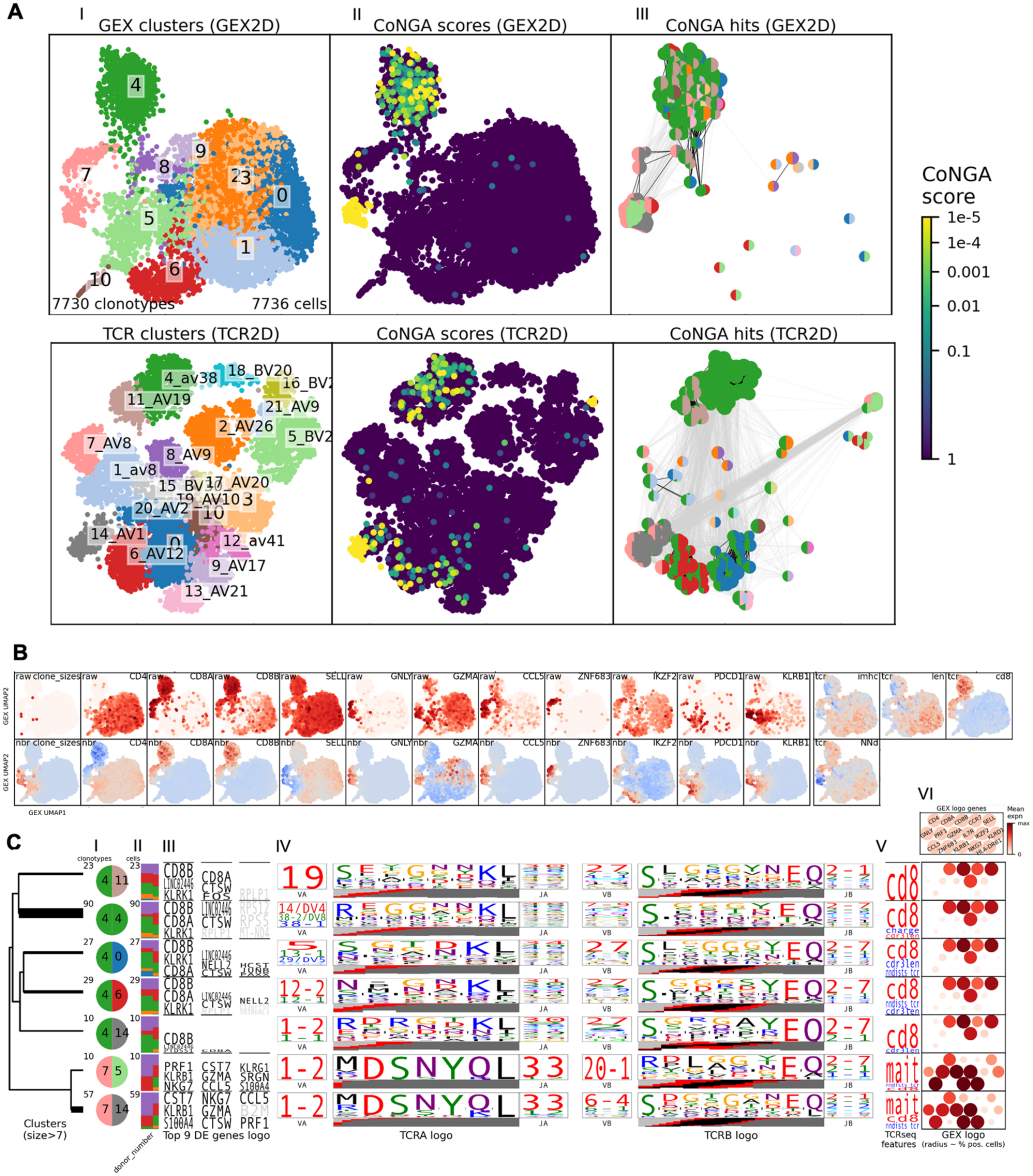
Clonotype Neighbour Graph Analysis (CoNGA) for identification of correlations between peripheral T cell gene expression (GEX) and T cell receptor (TCR) datasets (N = 5). (**A**) (I) UMAP projections coloured by GEX (top row) and TCR (bottom row) clusters. (II) UMAP projections coloured by “CoNGA scores”, indicating clonotypes residing in overlapping neighbourhoods in both the GEX and TCR datasets. (III) Bi-coloured discs indicating the GEX (left half) and TCR (right half) cluster assignment of clonotypes with low “CoNGA scores” (“CoNGA hits”). (**B**) Raw and Z-score normalised and GEX-neighbourhood averaged (nbr) expression of selected genes and TCR features across the thymic GEX UMAP. Blue colour indicates negative values, red colour indicates positive values. (**C**) Gene expression and TCR features among clonotypes grouped into “CoNGA clusters”. Shown are (I) bi-coloured discs indicating GEX (left half) and TCR (right half) cluster assignment, (II) donor origin of clonotypes, (III) differentially expressed genes, (IV) frequently used TCR gene segments and amino acid sequences, (V) properties of the TCR repertoire (represented as numerical TCR feature scores, red colour indicates increased scores, blue colour indicates decrease scores), and (VI) expression of selected genes.

We next applied CoNGA to the peripheral T cell TCR and GEX dataset (Fig. 4A–C, Supplementary Fig. 2B, Supplementary Fig. 6, Supplementary table 6). We observed clonotypes residing within overlapping neighbourhoods in the GEX and TCR datasets, indicated by low “CoNGA scores”, among CD8^+^ T cells (GEX clusters 4 and 7) (Fig. 4A). GEX cluster 7 expressed *CCL5* and consisted of *GNLY*^+^ and *ZNF683*^+^ subsets, with low “CoNGA scores” largely mapping to the *GNLY*^+^ subset (Fig. 4A, B). The clonotypes of GEX cluster 7 participated in two “CoNGA clusters” (7:5 and 7:14), which were marked by *TRAV1-2* and *TRAJ33* usage^37,38^ and a corresponding high score defining TCR sequences matching that of a consensus sequence for the receptor of mucosal-associated invariant T cells (“mait” score)^26^ (Fig. 4C, Supplementary table 6). Plotting on the GEX UMAP indicated that the mait cell-associated TCR sequences mainly related to the *GNLY*^+^-expressing subset (Supplementary Fig. 6, Supplementary table 6). Clonotypes of GEX cluster 7 exhibited enrichment for CDR3aa mediating strong interactions, indicated as a significant increase in the “strength” score (P_adj_ = 0.024), but not in the “imhc” score indicating *ZNF683*^+^-associated features. Further, we note that the increase in “strength” was also observed for GEX cluster 4 (P_adj_ = 4.7 × 10^–7^).

In sum, biases in the peripheral TCR repertoire were largely attributed to peripheral mait cells, with the *ZNF683*^+^ subset exhibiting no increase in the previously reported “imhc” score, in resemblance to the thymic *ZNF683*^+^ cells.

### *TRA* chains exhibit a higher degree of clonal overlap compared to *TRB* chains

**Figure 5:**
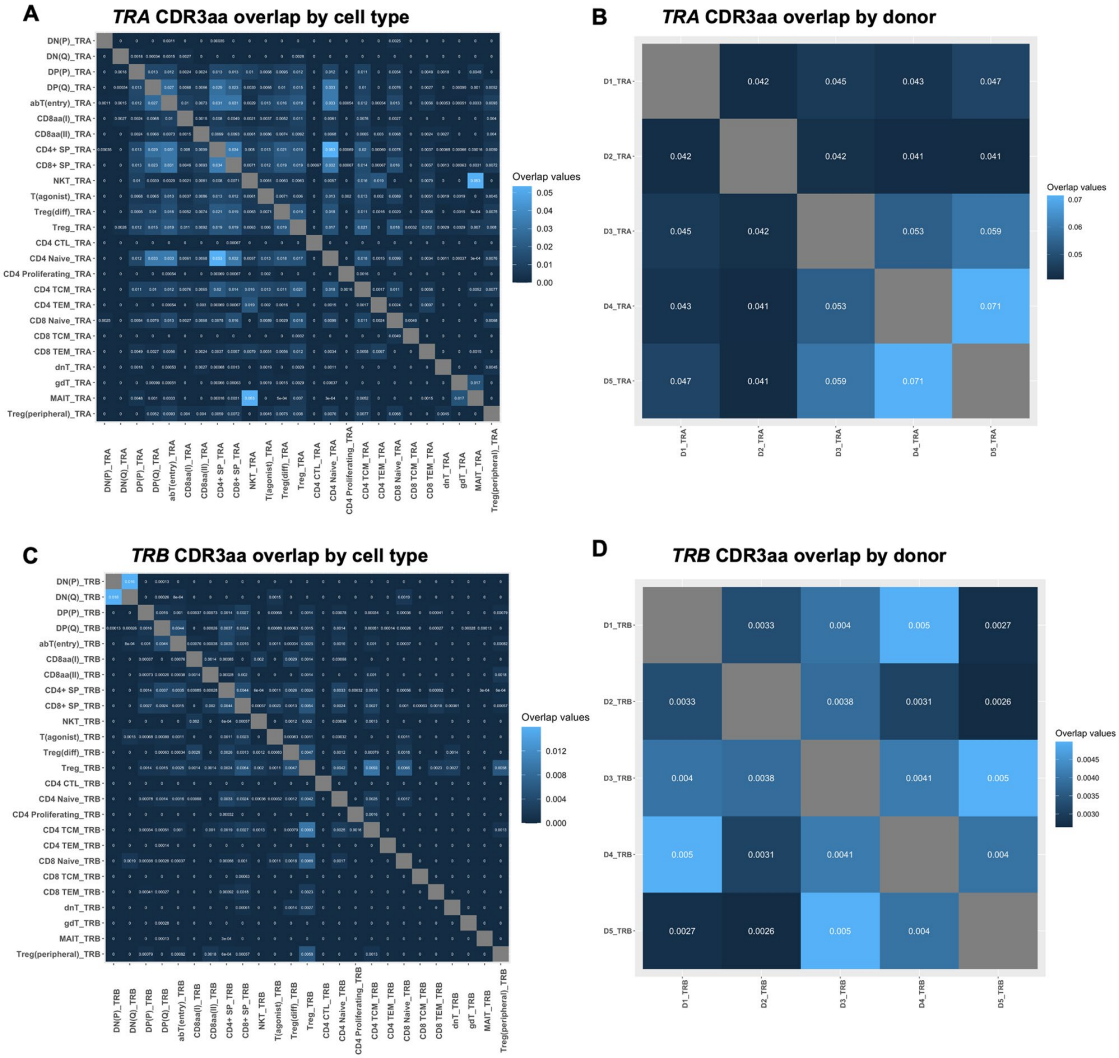
Clonal overlap in TCR data. Overlap (Morisita-Horn index) of unique *TRA* (top row) and *TRB* (bottom row) CDR3aa sequences among thymocytes and peripheral T cells across either cell types (left panels) or donors (right panels).

We next determined clonal overlap of *TRA* and *TRB* chains across donors and cell populations in thymus and blood, using the Morisita-Horn index as implemented in Immunarch (Fig. 5A–D). While the extent of overlap was limited, we observed a trend towards higher overlap for *TRA* compared to *TRB*, in agreement with previous reports^39^.

### Late stage thymocytes were enriched for pathology-specific CDR3aa *TRB* sequences

To identify pathology-associated CDR3aa sequences, we investigated overlap between our TCR data and CDR3aa sequences from the “autoimmune” or “pathogens” categories of the McPAS-TCR database^24^. *TRA* and *TRB* CDR3aa sequences were assessed separately for early stage thymocytes, agonist selected thymocytes, late stage thymocytes, peripheral CD4 T cells (CD4 CTL, CD4 naive, CD4 Proliferating, CD4 TCM, CD4 TEM), and peripheral CD8 T cells (CD8 naive, CD8 TCM, CD8 TEM) (Supplementary table 4)^25^. Enrichment of pathology-associated sequences was particularly pronounced for *TRB* sequences of late stage thymocytes, with significant (Bonferroni corrected Fisher’s exact t-test) enrichment of Influenza—(P_adj_ = 0.013), Celiac disease—(P_adj_ = 1.7 × 10^–4^), and Inflammatory bowel disease (IBD)—(P_adj_ = 5.0 × 10^–5^) associated sequences, implying the presence of both pathogen—and autoimmune-associated CDR3aa sequences among post-selection thymocytes.

## Discussion

In this work, we have performed scAIRR-seq on human thymus and blood from young paediatric donors, at an age prior to extensive thymic involution. We have previously reported GEX profiling of the same single cells, with cell type annotations^17^. Here we explored potential differences in the immune cell receptor repertoires across the identified cell populations. The use of single cell technology permitted coupling of immune receptor sequences to the transcriptional profile of each cell, facilitating assessment of TCR profiles across distinct thymic cell populations. Two distinct analytical approaches were used, where one was based on the detailed cell type annotations of the GEX dataset and the Immunarch workflow. The second approach was based on the CoNGA pipeline, which introduces a new integrative clustering using both gene expression and TCR data.

Both approaches identified dynamic changes in the TCR repertoire during thymocyte development. First, both approaches indicated expression of *TRA* as early as the DP(P) thymocyte stage, with a favouring of proximal *TRA* genes, before a shift towards distal *TRA* genes at the DP(Q) stage. This contrast with the established model of *TRA* recombination at the DP(Q) stage, and has previously been observed by others in human thymic single cell TCR sequencing data^14^. Previous work has revealed that premature *TRA* expression may occur in transgenic cell lines, resulting in expansion of CD8$\mathrm{\alpha \alpha }$ IELs^40^. Early *TRA* expression has also been reported in non-transgenic murine thymocytes^41^. However, these studies reported a preference for the distal *TRAV1* gene, potentially induced from the E$\updelta$ rather than the E$\mathrm{\alpha }$ enhancer, while we observed a preference for the proximal *TRA* gene. Again, our observations aligned with previous single cell human TCR data^14^. A preference for proximal *TRA* genes for more immature thymocyte populations, followed by a shift towards distal genes as development progresses, would also be in agreement with the suggested processive mechanism of repeated *TRA* recombination events^42^.

Both our analytical approaches further implied a shortening of the CDR3 region as thymocytes progress from DP to more mature stages, which potentially could be explained by biases inferred by selection events at the $\mathrm{\alpha \beta }$T(entry) stage^43^. As such, both pipelines resulted in similar observations consistent with previous literature, highlighting robustness across approaches.

For the more mature thymocytes and T cells, clonotypes that resided in overlapping neighbourhoods in the GEX and TCR datasets were largely attributed to CD8^+^ rather than CD4^+^ populations. This is in agreement with the previous CoNGA analyses of both PBMC and thymic samples^26^, potentially explained by a more restricted repertoire for CD8^+^ T cells compared to CD4^+^ T cells^44^. Alternatively, this could indicate an increased suitability for the CoNGA approach in identification of CD8^+^ compared to CD4^+^-derived TCR biases. Nevertheless, CoNGA appeared well suited for elucidation of the choice between conventional CD8^+^ SP selection or CD8$\mathrm{\alpha \alpha }$ lineage differentiation.

Previously, CoNGA has identified specific TCR features associated with thymic and peripheral *ZNF683*^+^ CD8$\mathrm{\alpha \alpha }$ T cells^14,26^, including CDR3aa sequences enriched for large, positively charged amino acids. However, we did not observe a significant increase in the “imhc” score, which summarises the *ZNF683*^+^-associated features, for the *ZNF683*^+^-expressing clusters in our dataset. We did, however, notice variation in several of the included TCR features among *ZNF683*^+^ cells, and we cannot exclude the possibility that the lack of a significant increase in “imhc” resulted from the limited data available.

Intriguingly, biases in TCR features were striking for a thymic *PDCD1*^+^ expressing cluster, including enrichment for large, positively charged amino acids associated with strong peptide-MHC:TCR interactions, and in this case resulting in a significantly upregulated “imhc” score. In our previous report, we observed a branch point between conventional and agonist selected thymocytes at a developmental time point prior to the SP stage, with the agonist selected lineages upregulating markers of a strong TCR response and signalling to APC subsets. The observation of *ZNF683*^+^-associated TCR biases in the *PDCD1*^+^ cluster, together with reports of a *PDCD1*^+^ precursor population for CD8$\mathrm{\alpha \alpha }$ T cells^31^, further supports this branch point. However, this must be corroborated by functional studies, and additionally, the role played by the developmental timing of *TRA* recombination for the choice between agonist or conventional positive selection needs further elucidation. We further note that the “imhc” score was upregulated also in DP thymocyte clusters, potentially implying that the TCR features reported to be associated with *ZNF683*^+^ T cells are also broadly present among less mature thymocyte stages or thymocytes that have not yet undergone conventional selection.

Plasticity between conventional and agonist selected lineages is exemplified by the ability of conventional, mature CD4^+^ T cells to differentiate to induced T_regs_ in the periphery. Induced T_regs_ appear to have a reduced lineage stability compared to thymically derived, natural T_regs_, an aspect that must be considered in the development of T_reg_-derived therapies^45–47^. Mirroring this plasticity, peripheral CD4^+^ T cells may also differentiate to become CD4^+^CD8$\mathrm{\alpha \alpha }$ IELs^48^. However, a model of TCR-specific biases for the *PDCD1*^+^ thymic population, suggested as a thymic CD8$\mathrm{\alpha \alpha }$ IEL precursor diverging prior to CD4^+^ lineage commitment, could imply cell-intrinsic differences between thymic-derived and peripherally induced CD8$\mathrm{\alpha \alpha }$ IELs, with particular implications in immune-mediated diseases in the gut such as IBD^9^. As such, further insights into differences between IELs of different developmental origin would increase the understanding of the role of the IEL compartment in immune regulation and disease.

Altogether, our study supported that *PDCD1*^+^ thymocytes exhibit a TCR repertoire bearing similarity to previously reported *ZNF683*^+^ populations, fitting with a branch point between conventional and agonist-selected thymocyte populations and potentially implying a developmentally timed role for the TCR in human CD8$\mathrm{\alpha \alpha }$ thymic T cell differentiation.

### Supplementary Information

Supplementary Information 1.

Supplementary Table 5.

Supplementary Table 6.

## Data Availability

Datasets are available from the Gene Expression Omnibus (GEO), accession number GSE227408*.*

## Abbreviations

aa: Amino acid
AIRR: Adaptive immune receptor repertoire
APC: Antigen presenting cell
BBKNN: Batch balanced K nearest neighbour
CDR3: Complementarity-determining region 3
CoNGA: Clonotype neighbour graph analysis
CTL: Cytotoxic T cell
DN(P): Proliferating CD4^-^CD8^-^ double negative
DN(Q): Quiescent CD4^-^CD8^-^ double negative
DP(P): Proliferating CD4^+^CD8^+^ double positive
DP(Q): Quiescent CD4^+^CD8^+^ double positive
GEX: Gene expression
IEL: Intraepithelial lymphocyte
imhc: Independent of peptide:MHC
mait: Mucosal-associated invariant T cell
MHC: Major histocompatibility complex
NKT: Natural killer T cell
P_adj_: Adjusted P value
PBMC: Peripheral blood mononuclear cells
scAIRR-seq: Single cell adaptive immune receptor repertoire sequencing
SP: CD4^+^CD8^-^ or CD4^-^CD8^+^ single positive
TCM: T central memory
TCR: T cell receptor
TEM: T effector memory
T_reg_: Regulatory T cell
UMAP: Uniform manifold approximation and projection
V(D)J recombination: Variable, (Diversity), and Joining gene segment recombination

## Code availability

Code central to the conclusions of the paper is available from Zenodo, https://doi.org/10.5281/zenodo.8260575. Additional code is available from the corresponding author upon request.

## References

[1] Krangel MS (2009). Mechanics of T cell receptor gene rearrangement. Curr. Opin. Immunol..

[2] Miho E (2018). Computational strategies for dissecting the high-dimensional complexity of adaptive immune repertoires. Front. Immunol..

[3] Wilson A, Held W, MacDonald HR (1994). Two waves of recombinase gene expression in developing thymocytes. J. Exp. Med..

[4] Irla M (2022). Instructive cues of thymic T cell selection. Annu. Rev. Immunol..

[5] Bouneaud C, Kourilsky P, Bousso P (2000). Impact of negative selection on the T cell repertoire reactive to a self-peptide: A large fraction of T cell clones escapes clonal deletion. Immunity.

[6] Yin Y, Li Y, Kerzic MC, Martin R, Mariuzza RA (2011). Structure of a TCR with high affinity for self-antigen reveals basis for escape from negative selection. EMBO J..

[7] Jordan MS (2001). Thymic selection of CD4+CD25+ regulatory T cells induced by an agonist self-peptide. Nat. Immunol..

[8] Bluestone JA (2015). Type 1 diabetes immunotherapy using polyclonal regulatory T cells. Sci Transl Med.

[9] Das G (2003). An important regulatory role for CD4+CD8 alpha alpha T cells in the intestinal epithelial layer in the prevention of inflammatory bowel disease. Proc. Natl. Acad. Sci. U. S. A..

[10] Ma H, Qiu Y, Yang H (2021). Intestinal intraepithelial lymphocytes: Maintainers of intestinal immune tolerance and regulators of intestinal immunity. J. Leukoc. Biol..

[11] Dijke IE (2016). Discarded human thymus is a novel source of stable and long-lived therapeutic regulatory T cells. Am. J. Transplant.

[12] Verstichel G (2017). The checkpoint for agonist selection precedes conventional selection in human thymus. Sci. Immunol..

[13] Morgana F (2022). Single-cell transcriptomics reveals discrete steps in regulatory T cell development in the human thymus. J. Immunol..

[14] Park, J. E. *et al.* A cell atlas of human thymic development defines T cell repertoire formation. *Science* **367**, eaay3224 (2020). 10.1126/science.aay3224 10.1126/science.aay3224 PMC7611066 32079746

[15] Kurd NS (2021). Factors that influence the thymic selection of CD8alphaalpha intraepithelial lymphocytes. Mucosal. Immunol..

[16] Leishman AJ (2002). Precursors of functional MHC class I- or class II-restricted CD8alphaalpha(+) T cells are positively selected in the thymus by agonist self-peptides. Immunity.

[17] Heimli M (2022). Multimodal human thymic profiling reveals trajectories and cellular milieu for T agonist selection. Front. Immunol..

[18] 10x Genomics. *Build notes for Reference Packages*. https://support.10xgenomics.com/single-cell-gene-expression/software/release-notes/build#GRCh38_2020A (2020).

[19] 10x Genomics. *Gene Expression Algorithms Overview*. https://support.10xgenomics.com/single-cell-gene-expression/software/pipelines/latest/algorithms/overview (2020).

[20] R Core Team. R: A language and environment for statistical computing. *R Foundation for Statistical Computing, Vienna, Austria*. https://www.R-project.org/, (2022).

[21] Nazarov, V. I. *et al.**Immunarch: Bioinformatics Analysis of T-Cell and B-Cell Immune Repertoires*. https://immunarch.com/, https://github.com/immunomind/immunarch, (2022).

[22] Kidman J (2020). Characteristics of TCR repertoire associated with successful immune checkpoint therapy responses. Front. Immunol..

[23] Rempala GA, Seweryn M (2013). Methods for diversity and overlap analysis in T-cell receptor populations. J. Math. Biol..

[24] Tickotsky N, Sagiv T, Prilusky J, Shifrut E, Friedman N (2017). McPAS-TCR: A manually curated catalogue of pathology-associated T cell receptor sequences. Bioinformatics.

[25] Amoriello R (2021). TCR repertoire diversity in Multiple Sclerosis: High-dimensional bioinformatics analysis of sequences from brain, cerebrospinal fluid and peripheral blood. EBioMedicine.

[26] Schattgen SA (2022). Integrating T cell receptor sequences and transcriptional profiles by clonotype neighbor graph analysis (CoNGA). Nat. Biotechnol..

[27] Polanski K (2020). BBKNN: Fast batch alignment of single cell transcriptomes. Bioinformatics.

[28] McInnes, L., Healy, J. & Melville, J. *UMAP: Uniform Manifold Approximation and Projection for Dimension Reduction*. 1802.03426v3 (2018).

[29] Traag VA, Waltman L, van Eck NJ (2019). From Louvain to Leiden: Guaranteeing well-connected communities. Sci. Rep..

[30] Wickham H, Sievert C (2016). Ggplot2: Elegant Graphics for Data Analysis.

[31] Ruscher R, Kummer RL, Lee YJ, Jameson SC, Hogquist KA (2017). CD8alphaalpha intraepithelial lymphocytes arise from two main thymic precursors. Nat. Immunol..

[32] Shugay M (2015). VDJtools: Unifying post-analysis of T cell receptor repertoires. PLoS Comput. Bio.l.

[33] Shugay, M. *vdjtools Documentation: Release snapshot*. https://readthedocs.org/projects/vdjtools-doc/downloads/pdf/master/ (2018).

[34] Rafei M (2011). Development and function of innate polyclonal TCRalphabeta+ CD8+ thymocytes. J. Immunol..

[35] Pita-Lopez ML, Pera A, Solana R (2016). Adaptive memory of human NK-like CD8(+) T-cells to aging, and viral and tumor antigens. Front. Immunol..

[36] Miyazawa S, Jernigan RL (1996). Residue-residue potentials with a favorable contact pair term and an unfavorable high packing density term, for simulation and threading. J. Mol. Biol..

[37] Tilloy F (1999). An invariant T cell receptor alpha chain defines a novel TAP-independent major histocompatibility complex class Ib-restricted alpha/beta T cell subpopulation in mammals. J. Exp. Med..

[38] Treiner E (2003). Selection of evolutionarily conserved mucosal-associated invariant T cells by MR1. Nature.

[39] Kitaura K, Shini T, Matsutani T, Suzuki R (2016). A new high-throughput sequencing method for determining diversity and similarity of T cell receptor (TCR) alpha and beta repertoires and identifying potential new invariant TCR alpha chains. BMC Immunol..

[40] Baldwin TA, Sandau MM, Jameson SC, Hogquist KA (2005). The timing of TCR alpha expression critically influences T cell development and selection. J. Exp. Med..

[41] Aifantis I (2006). The E delta enhancer controls the generation of CD4- CD8- alphabetaTCR-expressing T cells that can give rise to different lineages of alphabeta T cells. J. Exp. Med..

[42] Carico ZM, Roy-Choudhury K, Zhang B, Zhuang Y, Krangel MS (2017). Tcrd rearrangement redirects a processive Tcra recombination program to expand the Tcra repertoire. Cell Rep..

[43] Hou X (2019). Shorter TCR beta-chains are highly enriched during thymic selection and antigen-driven selection. Front. Immunol..

[44] Li HM (2016). TCRbeta repertoire of CD4+ and CD8+ T cells is distinct in richness, distribution, and CDR3 amino acid composition. J. Leukoc. Biol..

[45] Yadav M (2012). Neuropilin-1 distinguishes natural and inducible regulatory T cells among regulatory T cell subsets in vivo. J. Exp. Med..

[46] Koenecke C (2009). Alloantigen-specific de novo-induced Foxp3+ Treg revert in vivo and do not protect from experimental GVHD. Eur. J. Immunol..

[47] Kanamori M, Nakatsukasa H, Okada M, Lu Q, Yoshimura A (2016). Induced regulatory T cells: Their development, stability, and applications. Trends Immunol..

[48] Bilate AM (2020). T cell receptor is required for differentiation, but not maintenance, of intestinal CD4(+) intraepithelial lymphocytes. Immunity.

