# Supplementary material for: Human thymic putative CD8αα precursors exhibit a biased TCR repertoire in single cell AIRR-seq
Source: Sci Rep. 2023 Oct 18;13:17714. doi: 10.1038/s41598-023-44693-4 (PMC10584817; doi:10.1038/s41598-023-44693-4)
Supplement: Supplementary file 1 — Supplementary Information 1. [file 41598_2023_44693_MOESM1_ESM.pdf]

Supplementary information

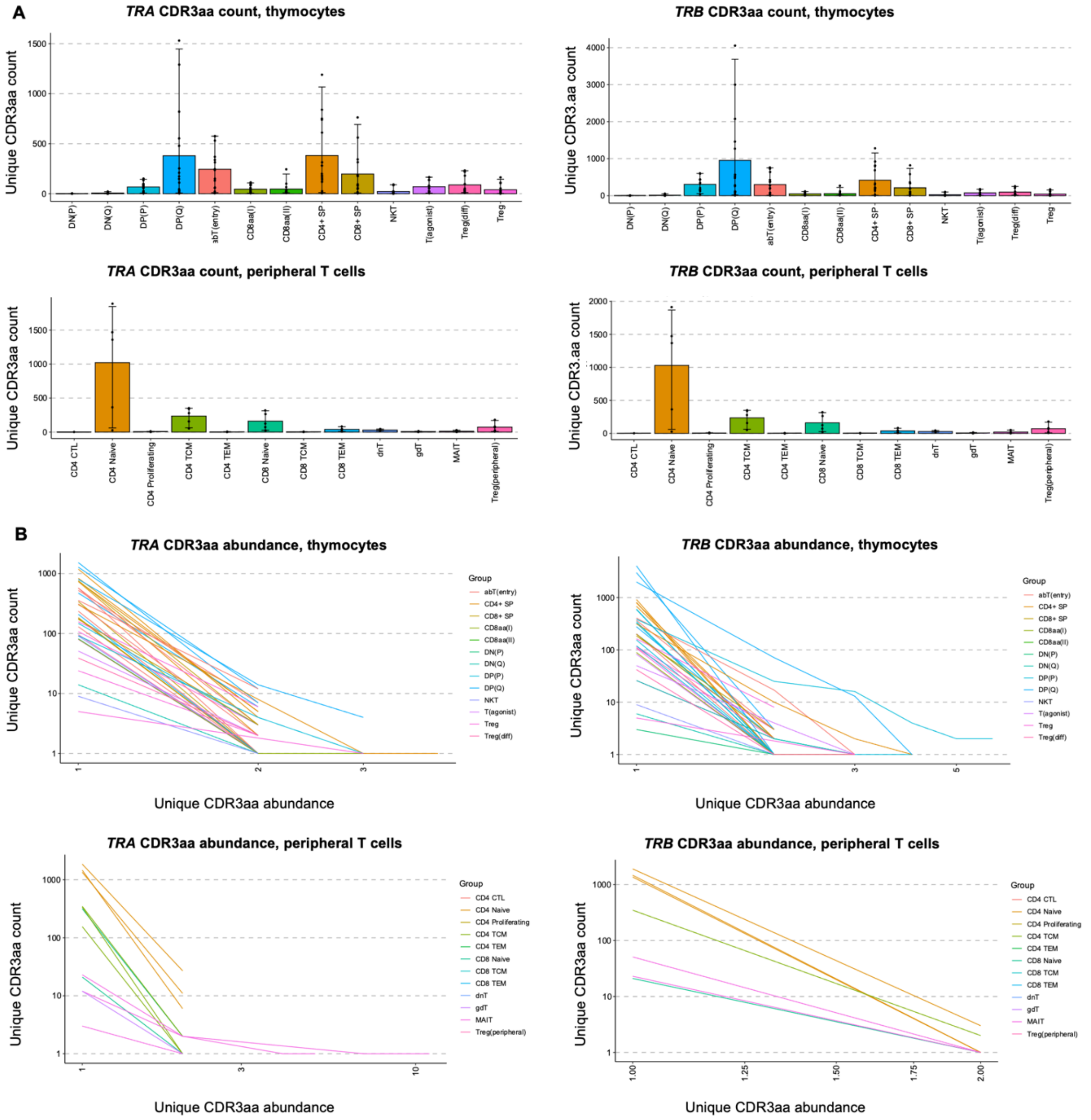

Supplementary figure 1: Counts and abundances of unique CDR3aa sequences in the TCR data. A. *Number of unique* TRA *and* TRB *CDR3aa sequences in thymocyte and peripheral T cell samples, grouped by cell type. Bar heights indicate means, lines indicate interquartile ranges, and dots indicate samples.* **B.** *Number of unique* TRA *and* TRB *CDR3aa sequences (y-axis) with a given abundance (number of occurrences of the unique CDR3aa, x-axis) for thymocyte and peripheral T cell samples. Lines indicate counts for one cell type in one sample, colour indicates cell type identity.*

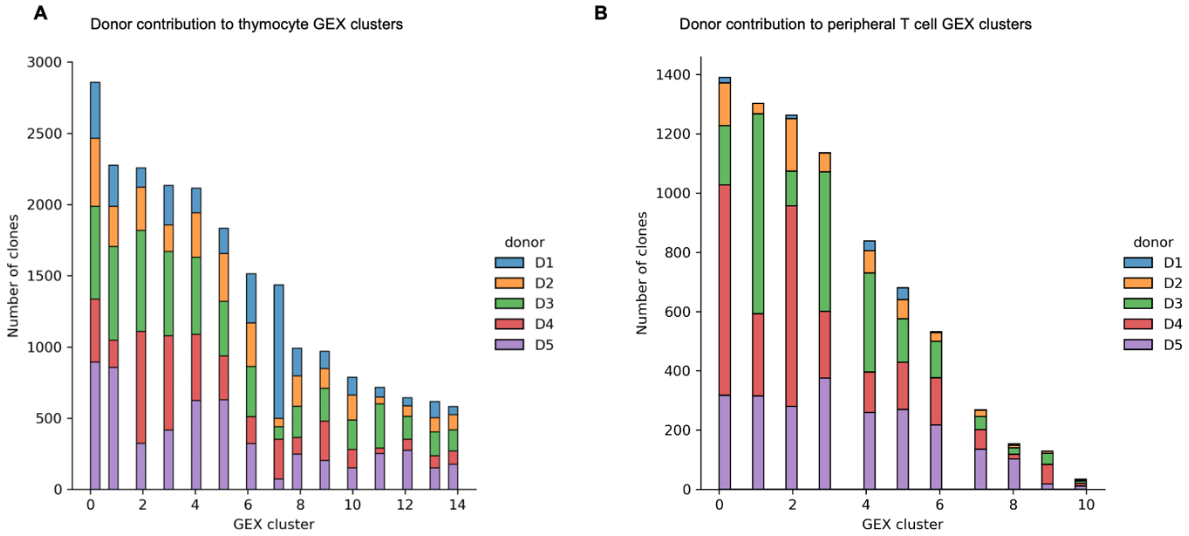

Supplementary figure 2: Donor origin of clonotypes in the CoNGA pipeline, across thymocyte and peripheral T cell GEX clusters.

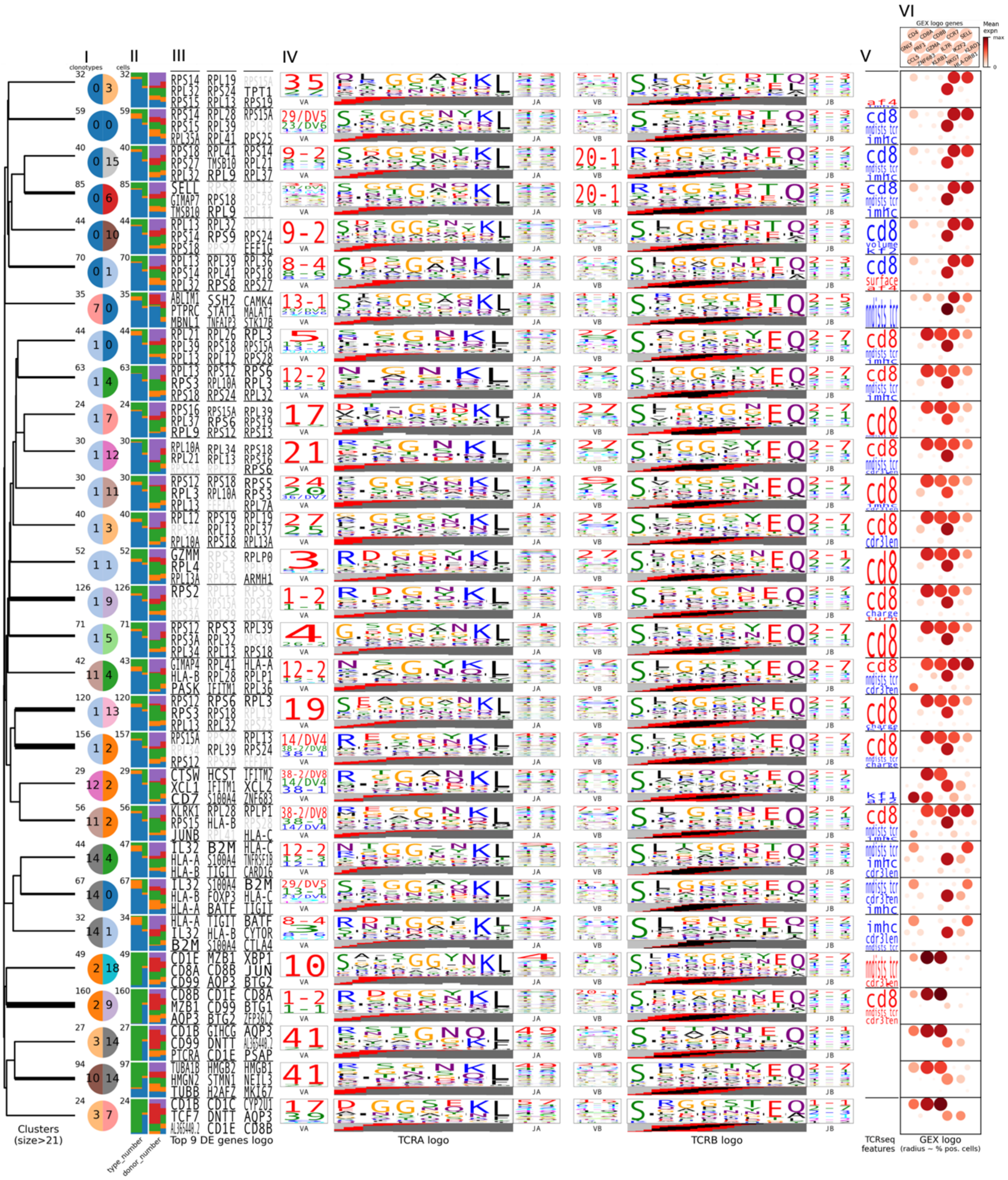
I
II
III
IV
V
VI
clonotypes
cells
GEX logo genes
Mean expn
max
0
Clusters
(size>21)
type_number
donor_number
Top 9 DE genes logo
TCRA logo
TCRB logo
TCRseq features
GEX logo (radius ~ % pos. cells)

Supplementary figure 3: "CoNGA clusters" representing overlaps between thymocyte GEX and TCR clusters. *Gene expression and TCR features among clonotypes grouped into "CoNGA clusters". Shown are (I) bi-coloured discs indicating GEX (left half) and TCR (right half) cluster assignment, (II) donor and sample type origin of clonotypes, (III) differentially expressed genes, (IV) frequently used TCR gene segments and amino acid sequences, (V) properties of the TCR repertoire (represented as numerical TCR feature scores, red colour indicates increased scores, blue colour indicates decrease scores), and (VI) expression of selected genes.*

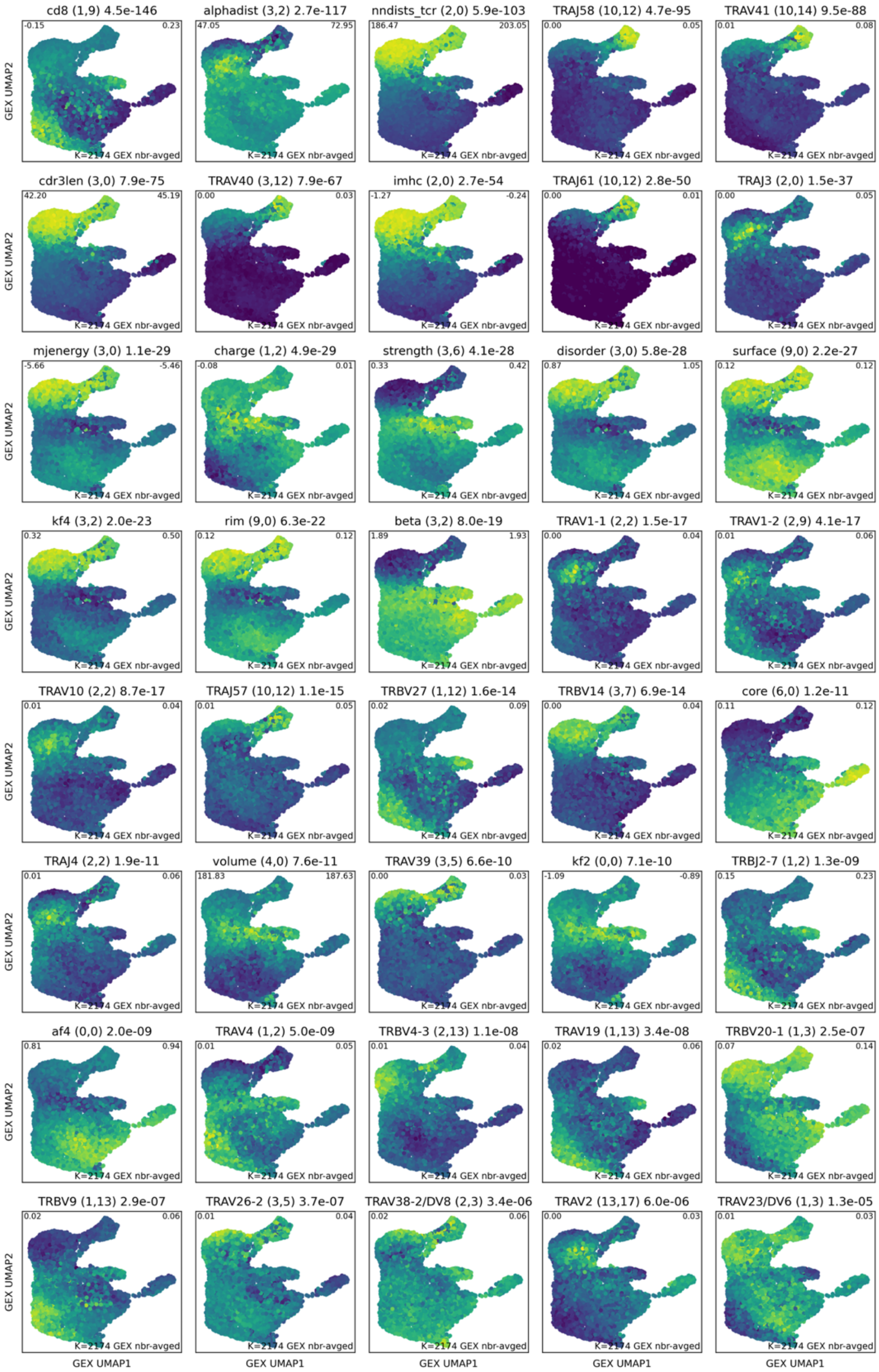
cd8 (1,9) 4.5e-146
alphadist (3,2) 2.7e-117
nndists_tcr (2,0) 5.9e-103
TRAJ58 (10,12) 4.7e-95
TRAV41 (10,14) 9.5e-88
cdr3len (3,0) 7.9e-75
TRAV40 (3,12) 7.9e-67
imhc (2,0) 2.7e-54
TRAJ61 (10,12) 2.8e-50
TRAJ3 (2,0) 1.5e-37
mjenergy (3,0) 1.1e-29
charge (1,2) 4.9e-29
strength (3,6) 4.1e-28
disorder (3,0) 5.8e-28
surface (9,0) 2.2e-27
kf4 (3,2) 2.0e-23
rim (9,0) 6.3e-22
beta (3,2) 8.0e-19
TRAV1-1 (2,2) 1.5e-17
TRAV1-2 (2,9) 4.1e-17
TRAV10 (2,2) 8.7e-17
TRAJ57 (10,12) 1.1e-15
TRBV27 (1,12) 1.6e-14
TRBV14 (3,7) 6.9e-14
core (6,0) 1.2e-11
TRAJ4 (2,2) 1.9e-11
volume (4,0) 7.6e-11
TRAV39 (3,5) 6.6e-10
kf2 (0,0) 7.1e-10
TRBJ2-7 (1,2) 1.3e-09
af4 (0,0) 2.0e-09
TRAV4 (1,2) 5.0e-09
TRBV4-3 (2,13) 1.1e-08
TRAV19 (1,13) 3.4e-08
TRBV20-1 (1,3) 2.5e-07
TRBV9 (1,13) 2.9e-07
TRAV26-2 (3,5) 3.7e-07
TRAV38-2/DV8 (2,3) 3.4e-06
TRAV2 (13,17) 6.0e-06
TRAV23/DV6 (1,3) 1.3e-05
K=2174 GEX nbr-avged
GEX UMAP2
GEX UMAP1

Supplementary figure 4: Highly variable TCR features across the thymocyte GEX dataset. *Numerical features indicating properties of the TCR repertoire are mapped onto both a GEX neighbourhood graph, and a GEX cluster graph where clonotypes residing in the same GEX cluster are connected. Variation in the TCR features across the GEX neighbourhood or cluster graph is assessed first by an initial t-test, then by a one-sided Mann-Whitney U test for the features with low t-test P values. Top scoring features, ranked by adjusted Mann-Whitney U test P values, are shown.*

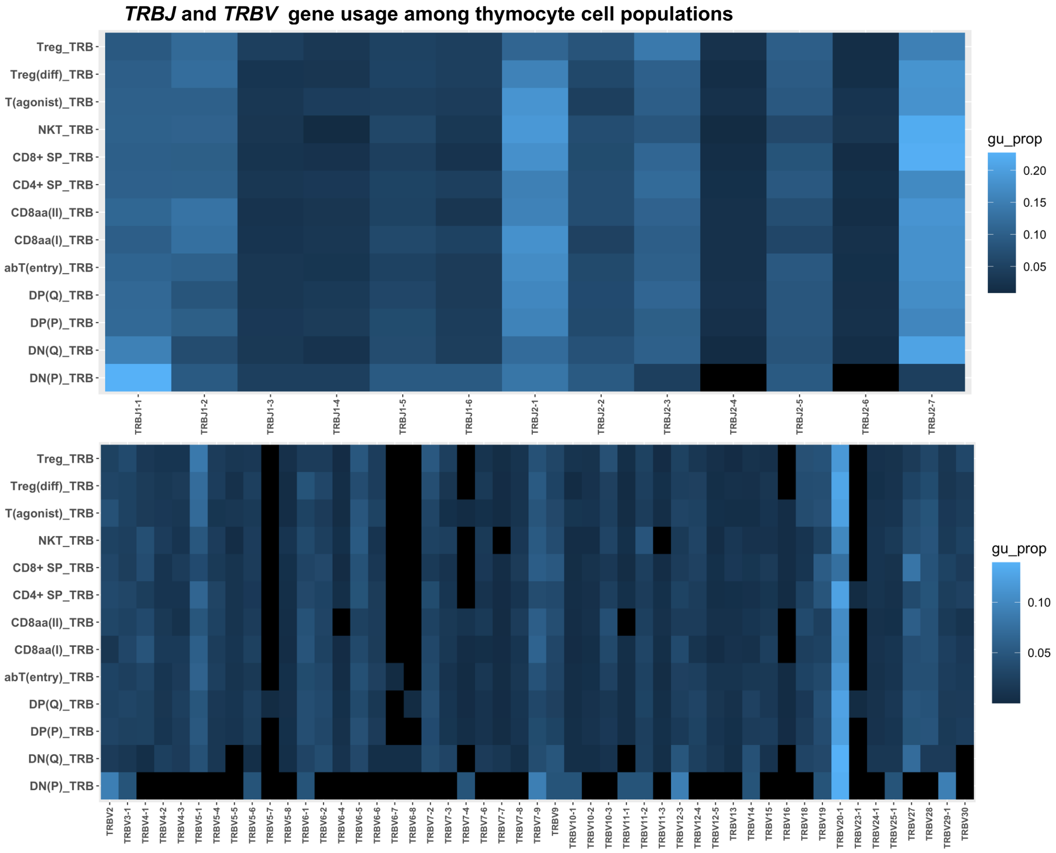

Supplementary figure 5: ***TRBJ* and *TRBV* gene usage across thymocyte populations.** *Colour indicates proportion among CellRanger-derived clonotypes weighted by clonal counts.*

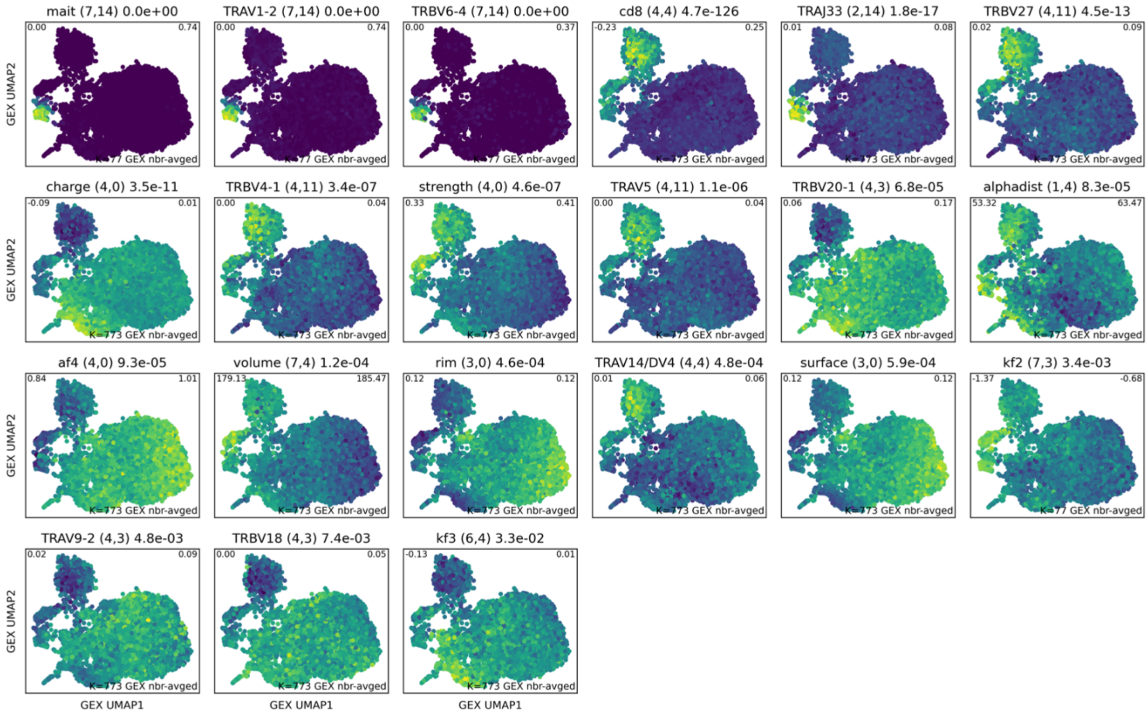

Supplementary figure 6: Highly variable TCR features across the peripheral T cell GEX dataset. *Shown are top scoring features after graph-versus-feature analysis of the peripheral T cell dataset, ranked by adjusted Mann-Whitney U test P values.*

Supplementary table 1: Donor metadata

Donor	Sex	Age
D1	Male	7 days
D2	Male	2.5 months
D3	Female	9 months
D4	Female	1.6 months
D5	Male	13.5 months

Supplementary table 2: CDR3aa lengths in thymocyte groups

Group	n (unique CDR3aa)	Mean length ± sd
Early_thymocytes_TRA	6711	14.05 ± 2.46
Agonist_thymocytes_TRA	4006	13.71 ± 1.84
Late_thymocytes_TRA	11876	13.75 ± 1.84
Early_thymocytes_TRB	19096	15.45 ± 2.14
Agonist_thymocytes_TRB	4402	14.60 ± 1.94
Late_thymocytes_TRB	13882	14.72 ± 1.94

Supplementary table 3: Welsh t-test of CDR3aa lengths between thymocyte groups

Comparison	t	df	P_{adj}	95% CI
Early_thymocytes_TRA vs. Late_thymocytes_TRA	8.61	11025	1.7×10^{-17}	[0.23, 0.36]
Early_thymocytes_TRA vs. Agonist_thymocytes_TRA	8.20	10170	5.2×10^{-16}	[0.26, 0.43]
Early_thymocytes_TRB vs. Late_thymocytes_TRB	31.93	31434	7.1×10^{-220}	[0.68, 0.77]
Early_thymocytes_TRB vs. Agonist_thymocytes_TRB	25.62	7085.7	2.9×10^{-138}	[0.78, 0.91]

Supplementary table 4: Fisher's exact t-test for enrichment of pathology-associated CDR3aa sequences

Group*	Pathology	Count	Odds ratio	P_{adj}
Agonist_thymocytes_TRA	Yellow fever virus	17	4.08	0.0018
Late_thymocytes_TRB	Influenza	38	2.26	0.013
Late_thymocytes_TRB	Celiac disease	10	8.96	1.7×10^{-4}
Late_thymocytes_TRB	Inflammatory bowel disease (IBD)	5	51.85	5.0×10^{-5}
CD4_T_TRB	Celiac disease	8	13.58	1.2×10^{-4}

**Group sizes: Early_thymocytes_TRA: 6711, Agonist_thymocytes_TRA: 4006, Late_thymocytes_TRA: 11876, Early_thymocytes_TRB: 19096, Agonist_thymocytes_TRB: 4402, Late_thymocytes_TRB: 13882, CD4_T_TRA: 6171, CD8_T_TRB: 1009, CD4_T_TRB: 6356, CD8_T_TRB: 1011*

Supplementary .csv table 5: Mann-Whitney U test for variation of TCR features across the thymocyte GEX cluster graph

Supplementary .csv table 6: Mann-Whitney U test for variation of TCR features across the peripheral T cell GEX cluster graph
